# Metabolic profiling and microbiological evaluation of *Staphylococcus aureus* in a poly-microbial system in soft white cheese

**DOI:** 10.1038/s41598-026-59959-w

**Published:** 2026-07-04

**Authors:** Mohamed F. El-Ssayad, M. Elaaser, Sally I. Abd-Elfatah, Salah H. Salem

**Affiliations:** 1 Dairy Sciences Department, National Research Centre, 33 El-Bohouth St, P.O. Box: 12622, Dokki, Cairo Egypt; 2 Food Toxicology and Contaminants Department, National Research Centre, 33 El-Bohouth St, P.O. Box: 12622, Dokki, Cairo Egypt

**Keywords:** Microbial interactions, *S. aureus*, GC-MS metabolomics, Foodborne pathogens, Nisin, EDTA, Soft cheese preservation, Biological techniques, Biotechnology, Microbiology

## Abstract

There is a significant lack of data on microbial interactions in polymicrobial food systems. This study aimed to investigate the effects of *Escherichia coli* and *Bacillus cereus* on *Staphylococcus aureus* growth and metabolite production in soft white cheese, a food model. The growth dynamics of single and mixed cultures of these pathogens in various microbial systems were examined, including single, binary, and tertiary combinations. Gas chromatography-mass spectrometry (GC-MS) was used to analyze the metabolic profiles of these poly-microbial systems. Additionally, we assessed the impact of nisin/EDTA on the tertiary microbial system in soft white cheese and evaluated its microbiological safety and quality. All microbiological experiments were performed in triplicates and data expressed as mean ± Std (*P* < 0.05). The results showed that *B. cereus* growth was significantly reduced in binary (*Bc -Sa*) and tertiary (*Bc- Sa-Ec*) systems, with reductions of 95.91% (1.42 Log cycle) and 94.77% (1.4 Log cycle), respectively. *E. coli* growth was also reduced in binary (*Ec- Sa*) and tertiary (*Bc- Sa-Ec*) systems, with reductions of 66.67% (0.48 Log cycle) and 96.67% (1.48 Log cycle), respectively. *S. aureus* growth was inhibited by 54.17% (0.33 Log cycle), 79.17% (0.67 Log cycle), and 91.67% (1.07 Log cycle) in (*Bc -Sa*), (*Bc- Sa-Ec*), and (*Ec- Sa*) systems, respectively. GC-MS analysis revealed the presence of various metabolites, including hydroxyurea, methane nitroso, and pyridinium derivatives, with different ratios depending on the poly-microbial system. The addition of nisin/EDTA effectively reduced growth in both single and poly-microbial systems, and its application in soft white cheese enhanced microbiological safety and quality. Our findings suggest that studying microbial interactions and metabolite production in poly-microbial systems can inform the development of effective preservation methods that improve food safety and quality while enhancing sensory characteristics.

## Introduction

Food microbiology is a vibrant field that explores the multifaceted roles of microorganisms in food, with a particular emphasis on their impact on food quality, safety, and nutritional value. The intricate relationships between microbes in food ecosystems play a pivotal role in determining the fate of food products, influencing factors such as spoilage, shelf life, and nutrient content. Elucidating these complex interactions is essential for the development of innovative food preservation strategies, optimized flavor profiles, and robust food safety protocols, ultimately yielding benefits for both consumers and producers^1,2^. Interactions among various sets of heterogeneous microbial populations and with their environment were reported in the food system^3^. Also, the food composition and its physical state are the main factors that determine the growth of spoilage and poisoning organisms in food^4^. Competence for substrates through production of inhibitory metabolites can change the local environment or directly affect the other competitors^5^. *S. aureus*; the foodborne toxigenic, gram-positive and non-spore-forming bacterial species that exists among normal skin flora^6^, resembles a common threat in different categories of food with high incidence^7^. Ingestion of the preformed Staphylococcal enterotoxin (s) (SETs) results in severe foodborne outbreaks worldwide^8^. Investigations of such accidents attributed the majority of these to poor practices, especially improper handling in retail places^9^, as well as poor cleaning and disinfection^10^, which often contribute to post-processing contamination of ready-to-eat foods. Such a foodborne microorganism, when it interacts with others was referred to be dynamic and complex with some implications on health and sustainability^11^. Earlier reports examined the potential metabolic behavior of certain fermentative flora in carbohydrate-rich media to ensure their nutrient security^12^. Another recent study examined the stability of microbial biofilms in both mono- and dual-culture systems after the addition of disinfectants^13^. Recently, the use of metabolic analyses to estimate microbial metabolites^14^ and microbial interactions has contributed greatly to the stability of foods^15^.

Previous studies have widely utilized *S. aureus* incidence, distribution, antibiotic resistance, toxin-producing potential^16^, and relation to other human diseases in clinical history^17^, but there is no sufficient background about the inter-relationships among different species, such as which particular microorganism affects or is affected in the presence of others regarding the safety of the food. This study was designed to determine whether *S. aureus* is enhanced or inhibited by the presence of both *Escherichia coli* and *Bacillus cereus*, as well as the consequences of this response for the metabolites produced. As well as how the suggested preservative, Nisin/EDTA, can affect *S. aureus* responses in the absence and presence of the other co-cultures.

## Methodology

### Growing the pathogenic bacteria

*Escherichia coli* (*E. coli*) strain E11 (accession number KY780346.1) and *Bacillus cereus* (*B. cereus*) strain 151,007-R3-K09-40–27 F (accession number KY820914.1) were isolated and identified by Al-Gamal et al.^18^, and *Staphylococcus aureus* (*S. aureus*) ATCC 6538 was obtained from the culture collection of Dairy science department (Microbiological laboratory), NRC. Each strain was grown in nutrient broth (Himedia, India) at 35 °C for 24 h.

### Estimation of bacterial count

Growth of the single cultures was estimated on plate count agar medium (Condalab., Spain) as directed by APHA^19^ for aerobic mesophilic bacterial count. Within poly microbial systems (*Sa-Bc*), (*Sa-Ec -Bc*), and (*Sa-Ec*); *S. aureus* growth was estimated on Baird-Parker agar (Merk, Germany) as reported by Baird-Parker^20^, *Escherichia coli* was counted as the total coliforms on Violet Red Bile agar according to the Bacteriological analytical Manual^21^, while *B. cereus* count was estimated on *B. cereus* (PEMBA) agar as recommended by Holbrook and Anderson^22^. Using the spread plating of 1 mL from each dilution on three plates, the limit of 1 Log CFU can be detected.

### GC/MS-based metabolic profiling of bacterial pathogens

Twenty-four-hour-old cultures of single and mixed cultures in binary (*Sa-Ec*), (*Sa-Bc*), or tertiary (*Sa-Bc-Ec*) polymicrobial systems were subjected to liquid-liquid extraction with ethyl acetate^23^. Ethyl acetate extracts were analyzed in the central Lab. Network, NRC, Egypt. For GC/MS analysis (GC-MS system [7890 A-5975 C, Agilent Technologies Inc., Santa Rosa, CA, USA]), 1 µl of Extract was injected in the presence of Helium as the carrier gas with flow rate of 1 ml/ minute following the same thermal program that used by Sharaf et al.^18^.

### Studying the effect of Nisin/ EDTA on the poly-microbial system

Estimating the effect of nisin/ EDTA on the growth of pathogenic strains; Nisin 100 ppm and EDTA 0.4% were added to Nutrient broth tubes of single, binary and tertiary microbial systems^24^. After 12 h incubation of the inoculated tubes (10^3^ CFU/mL). The inoculum was standardized by adjusting the absorbance at 625 nm to 0.08 (EUCAST^25^), equivalent to 1 × 10^8^ CFU/mL, and then serially diluted. The viable count of each pathogen was assessed on the recommended selective agar media, and the count was recorded as Log CFU/mL. The limit of microbial detection is 1 Log CFU/ mL.

### In-*Situ* experiment to evaluate the role of Nisin/EDTA in a real dairy product

Microbiological status was assessed in the tertiary state (*Sa-Bc-Ec*) to evaluate the effects of Nisin 100 ppm^26^ and EDTA 0.4%^24^ on the bacterial pathogens studied. According to Mehaia^27^, two batches of white soft cheese were manufactured; one batch without the addition of preservative (Untreated group), while the other was supplemented with preservative at the recommended concentrations (Nisin/EDTA-treated group).

### Assessing the microbiological safety and quality of cheese

Microbiological examinations to evaluate safety and quality were performed. Aerobic mesophilic bacterial count^19,21^, *Escherichia coli*^28^, and detection of *Salmonella *were assayed according to APHA^19^; FDA^21^, while Molds and yeasts were enumerated as recommended by Galloway and Burgess^29^. All the microbial counts were performed in triplicates.

### Statistical analysis

Statistical significance was determined using Minitab 18. Analysis of variance test (ANOVA, two-way analysis) was performed (*P* < 0.05) and means were determined.

## Results

### Impact of Poly-microbial colonization on the recovered cell growth

**Fig. 1:**
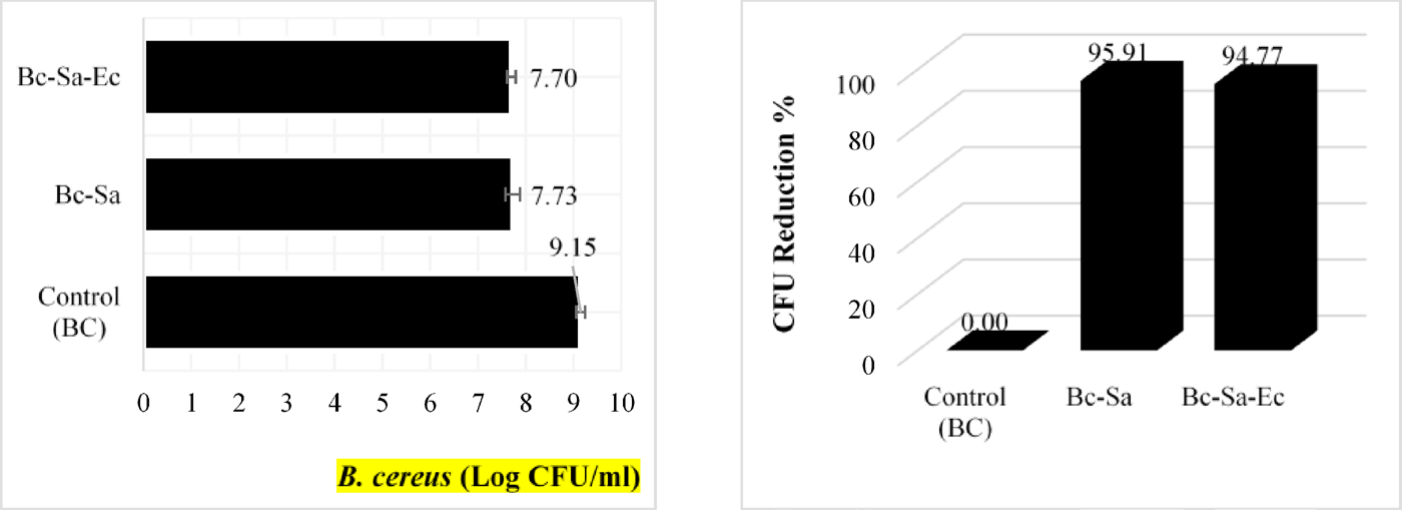
Effect of the Poly-microbial system on ***B. cereus*** growth.

Studying the Poly-microbial system (PMS), the investigation starts with the effect of interaction on the growth and development of *S. aureus*, as well as the effect of *S. aureus* on both *B. cereus* and *E. coli*. Figure 1 presents how *B. cereus* growth is affected when included either in a binary or in a tertiary microbial system. After incubation, *B. cereus* as a single culture recorded 1.47 × 10^9^ CFU/ mL in the basic broth medium. Combining *B. cereus* with *S. aureus* (*Bc-Sa*) decreased the count *B. cereus* to 6.0 × 10^7^ CFU/ mL. This decrease represents 95.91% of the single-cultured *B. cereus*. Furthermore, the viable cell number of *B. cereus* slightly increased to 7.67 × 10^7^ CFU/ mL achieving a reduction of 94.77% of the single-cultured *B. cereus* in the tertiary system (*Bc-Sa-Ec*).

**Fig. 2:**
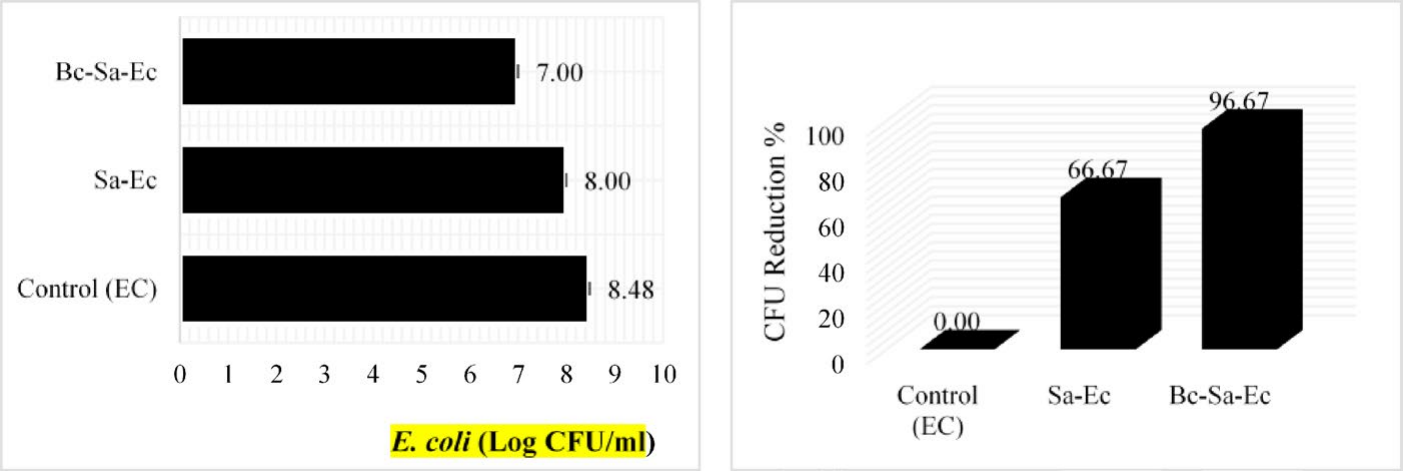
Effect of the Poly-microbial system on ***E. coli*** growth.

Growth and development of the gram-negative bacterium; *Escherichia coli* was also studied. Figure 2 shows how *Escherichia coli* impacted with *S. aureus* growth in a binary or a tertiary system. Number of the uncombined *E. coli* maximally reached 30 × 10^7^ CFU/ ml. In the *S. aureus*-based binary system (*Sa-Ec*), *E. coli* numbers were decreased to 10 × 10^7^ CFU/ mL losing 66.67% of their density. Moreover, the tertiary microbial system; *E. coli* with both of *S. aureus* and *B. cereus* (*Bc-Sa-Ec*) contributed to the enteric count reduction to 1 × 10^7^ CFU/ mL removing 96.67% of *E. coli* population.

**Fig. 3:**
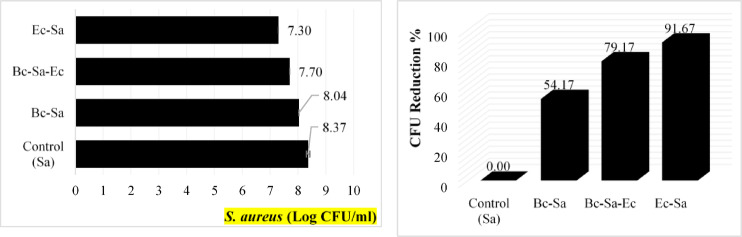
The estimated growth of *S. aureus* in a poly-microbial system.

The growth of *S. aureus* was also tracked to evaluate the effect of inclusion either in a binary system with *B. cereus* (*Bc-Sa*) or with *E. coli* (*Ec-Sa*) or in a tertiary system; with both *B. cereus* and *E. coli* (*Bc-Sa-Ec*). Cell number of *S. aureus* was also followed in a single state (control), binary states; the 1st when combined with *B. cereus* and the other with *E. coli*, and in the tertiary system with both *B. cereus* and *E. coli* and results were shown in fig. 3.

The single-cultured *S. aureus* recorded the maximum density of 24 × 10^7^ CFU/ mL. The count was lowered to 10 × 10^7^ CFU/ mL losing 54.17% of cell density as a result of *B. cereus*-based combination. The *E. coli*-based combination reduced *S. aureus* population to 2 × 10^7^ CFU/ mL raising growth reduction to 91.67%. When *S. aureus* was included in the tertiary system, its density was re-increased to 5 × 10^7^ CFU/ mL that subsequently recovered growth and held the cell lose at 79.17%.

### The influence of poly-microbial system on the metabolic response

Production of the listed metabolites are appeared to be stimulated by the presence of *S. aureus*. Volatile metabolites were estimated by the Headspace-GC/MS analysis, and results were collected in Table (1).

1,1-bis (bromanyl)-2-chloranyl-2-fluoranyl-cyclopropane was the first detected (RT: 2.029–2.071 min). This derivative- as shown- was only produced by the tertiary poly-microbial culture (*Bc-Sa-Ec*) representing about 55% of the extract. Another derivative; the Propylcyclobutane was produced by *S. aureus*/ *E. coli* – based combinations. In a *Sa-Ec* binary system, the produced Propylcyclobutane was 12.21%, while the inclusion of *B. cereus* resulted in increased production (33.78%) by the BES system. Cyclopropane derivatives were known to be synthesized by microbes^30,31^ and their antibacterial activity was reported by Salaün^32^.

**Table 1:** GC/MS-estimated metabolic response of the S. aureus–based poly–microbial systems.

NO	RT	Name	Chemical formula	The % Incidence of chemical compounds in single and different Poly-microbial combinations					
				Sa	Bc	Ec	Bc-Ec-Sa	Bc-Sa	Ec-Sa
1	2.029–2.071	1,1-bis(bromanyl)-2-chloranyl-2-fluoranyl-cyclopropane;	C3H2Br2ClF	ND	ND	ND	55.2	ND	ND
2	2.47–2.57	Hydroxyurea	CH4N2O2	1.71	0.14	0.27	ND	0.65	5.22
3	2.643	Methane, nitroso-	CH3NO	ND	ND	ND	ND	8.28	5.42
4	2.727–2.824	Methylazoxymethanol acetate	C4H8N2O3	ND	ND	ND	ND	9.58	ND
5	2.951	Ethyl Acetate	C4H8O2	ND	ND	ND	9.43	52.25	1.75
6	3.09	Methanamine, N-methyl-, compd. with borane (1:1)	C2H10BN	ND	11.88	ND	ND	0.36	ND
7	3.116–3.192	Formamidine acetate	C3H8N2O2	ND	ND	2.24	ND	ND	ND
9	4.405–4.718	Propane, 2-isocyanato-	C4H7NO	ND	1.82	2.25	ND	ND	ND
10	4.93	Propylcyclobutane	C7H14	ND	ND	ND	33.78	ND	12.21
12	10.655–10.956	1-(1-Carboxyethyl) pyridinium	C8H9NO2	2.29	4.0	0.94	ND	ND	ND
13	10.896	Pyrazine, 2,5-dimethyl-	C6H8N2	ND	ND	ND	1.27	13.68	7.65
14	14.174	2,5-Diaminotoluene sulfate	C7H12N2O4S	ND	ND	ND	0.14	ND	1.16
15	16.622–16.745	3-Azidophenol	C6H5N3O	0.81	ND	ND	0.11	ND	ND
16	16.618–16.716	(9-(2’,3’-dideoxy-.beta.-D-glucopyranosyl)-adenine	C11H15N5O3	ND	0.69	ND	ND	1.18	0.77
17	21.07	Carvone	C10H14O	12.24	ND	ND	ND	ND	ND
18	22.98	Phenol, diethyl-	C10H14O	9.87	ND	ND	ND	ND	ND
19	23.024–23.447	[(E)-2-azidoethenyl] benzene	C8H7N3	ND	ND	1.67	ND	ND	0.56
20	28.4	Butylated hydroxy toluene	C15H24O	25.75	13.59	10.25	ND	ND	ND
21	28.750	Benzoic acid, 4-ethoxy-, ethyl ester;	C11H14O3	3.11	ND	ND	ND	ND	ND

*Sa*: *S. aureus*; ***Bc***: *B. cereus*; *Ec*: *E. coli*; *Bc-Ec-Sa*: *B. cereus* + *E. coli* + *S. aureus*.

Hydroxyurea (HU) that appeared after 2.47–2.57 min. was produced minimally by the single system of *B. cereus* (0.14%), moderately by *E. coli* (0.27%), and maximally by *S. aureus* (1.71%). Binary systems affected the production of Hydroxyurea. In *Bc-Sa* system, HU was reduced to 0.65%, while *Ec-Sa* culture increased the produced HU to 5.22%. Subjecting of *S. aureus* in a Tertiary (BES) microbial system completely inhibited the production of HU. However, there is no available research confirms the microbial synthesis of hydroxyurea, this compound and derivatives as possible antimicrobial agents were reported^33,34^. Furthermore, some derivatives were confirmed for their bacteriostatic activity against *E. coli*^35^.

Methane nitroso was detected at 2.643 min. only in binary systems; *Sa-Ec* samples (5.42%) and increased in *Bc-Sa* samples to (8.28%). Neither single cultures nor the tertiary microbial system could produce this compound. This and such compounds are applied in bacterial disinfection and biocontrol of bacterial biofilms^36^ through inducing cell damage that based on reactive oxygen radicals^37^.

Methylazoxymethanol acetate (RT: 2.727–2.824 min) was appeared only in the dual culture of *B. cereus*/*S. aureus* (*Sa-Bc*) at 9.58%. This compound was reported as a potent colon cancer agent^38^.

Methylamine derivatives that detected at RT of 3.09 min. were mainly produced by *B. cereus* culture with maximum concentration of 11.88%. Introducing *S. aureus* in combination with *B. cereus* (*Bc-Sa*) severely decreased the produced Methylamine to 0.36%. Many compounds of important clinical properties are based on methylamine nucleus. Some of these synthesized compounds recorded a considerable activity against both bacteria and fungi^39^.

Pyridinium derivatives (1-(1-Carboxyethyl) pyridinium) that produced at RT of 10.655–10.956 min. was only detected in samples of single-cultured systems as 0.94% (*E. coli*), 2.29% (*S. aureus*), and 4% (*B. cereus*). Pyridinium derivatives neither produced by binary systems nor by the tertiary one. Recent articles^40^ reviewed that pyridine-derived formulas possess a wide-spectrum inhibition toward gram positive bacteria (*B. subtilis* and *S. aureus*), gram negative bacteria (*E. coli*), mold (*A. niger*), and yeast (*C. albicans*).

2,5-Dimethyl Pyrazine that detected at 10.896 min. was produced all *S. aureus* – based mixtures; *Sa-Bc* (13.68%), reduced to and 7.65% within *Sa-Ec*, and reduced more to 1.27% within BES system. Also, Alkyl pyrazines exhibited a wide range of biological activities^41^, but the 2,5-dimethyl derivative contribute to bacterial growth (especially *E. coli*) inhibition^42^.

Carvone (RT: 21.07 min.); the monoterpene ketone that is detected only in the single-cultured *S. aureus* sample in 12.24%. It is commonly synthesized via microbial transformations^43^ and possesses a good antimicrobial activity against *E. coli* and *candida spp*.^44^.

Azidoethenyl Benzene does not have enough results that support the possible antibacterial contribution^45^.

Diethyl phenol (RT: 22.98 min); the phenolic lipid that only detected in the single-cultured *S. aureus* samples in 9.78%. Phenolic lipids, as well as phenolic compounds which derive from mono and dihydroxy phenols were widely reviewed as strong antimicrobial^46^ and antibiofilm agents^47^.

Butylated Hydroxy Toluene (BHT) was detected at 28.04 min in samples of all single-cultured pathogen at rates of 10.25% (*E. coli*), 13.59% (*B. cereus*), and 25.75% (*S. aureus*). This lipophilic antioxidant is commonly applied as a food additive with high antioxidant activity^48^ and sometimes as a preservative that indirectly prevents food spoilage through increasing the food resistance to pathogen infection^49^.

Benzoic acid-4-ethoxyethyl ester; the ethyl ester of benzoic acid (RT: 3.11 min) was detected only in the single-cultured *S. aureus* samples at 3.11%. Benzoic acid esters possess a good, but narrow antibacterial effect especially against gram positive and *S. aureus*^50^. Several studies reported the possibility of ethyl-4-ethoxybenzoate biosynthesis by bacteria^51^.

### Influence of Nisin/EDTA on the recovered pathogens growth with Poly-microbial system

**Fig. 4:**
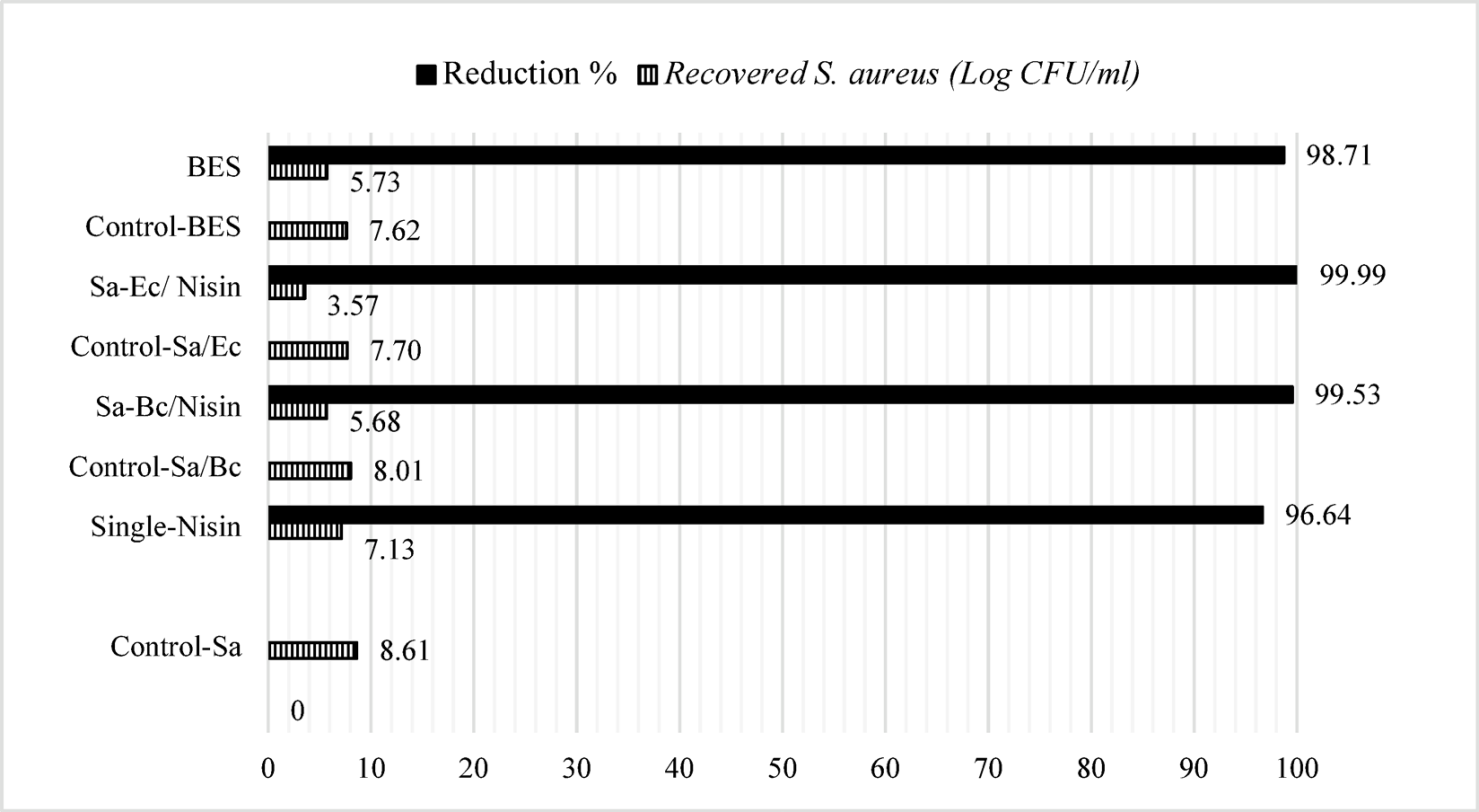
Influence of Nisin/ EDTA on the poly-microbial- included *S. aureus*.

Estimating the effect of nisin (100 ppm) and EDTA (0.4%), each one of the three utilized pathogens; *S. aureus*, *B. cereus*, and *E. coli* was analyzed in the single state without addition of preservative (Control), as single with preservation, in a binary state with preservation, and in the tertiary state (*Sa-Bc-Ec*) with preservation. The response of *S. aureus* was presented in fig. 4. Addition of Nisin/ EDTA as preservative ensured to significantly affect the growth of *S. aureus* populations either when single-grown or combined in a poly-microbial community (*P* < 0.05). Without addition of the preservative, the maximum *S. aureus* growth was estimated as 8.61 Log CFU/ mL, but Introduction of Nisin/ EDTA lowered the *S. aureus* growth to 7.13 Log CFU/ mL achieving 96.64% growth reduction. Within *B. cereus*-based combinations (*Bc-Sa*/ Nisin), *S. aureus* growth lowered from 8.01 to 5.68 Log CFU/ mL achieving growth reduction by 99.53%. The maximum reduction of *S. aureus* growth (4.13 Log cycles) was observed in *E. coli*-based combination (*Sa-Ec*/ Nisin). The preservative; Nisin/ EDTA caused drop in *S. aureus* growth from 7.70 to 3.57 Log CFU/ mL (4.13 Log cycles) increasing the growth reduction to 99.99%. In the tertiary (*Sa-Bc-Ec*) system, Nisin/ EDTA retained a limited effect against *S. aureus* growth. It just lowered the count of S. *aureus* from 7.62 to 5.73 Log CFU/ mL (1.89 Log cycles) reducing the growth by 98.71%.

**Fig. 5:**
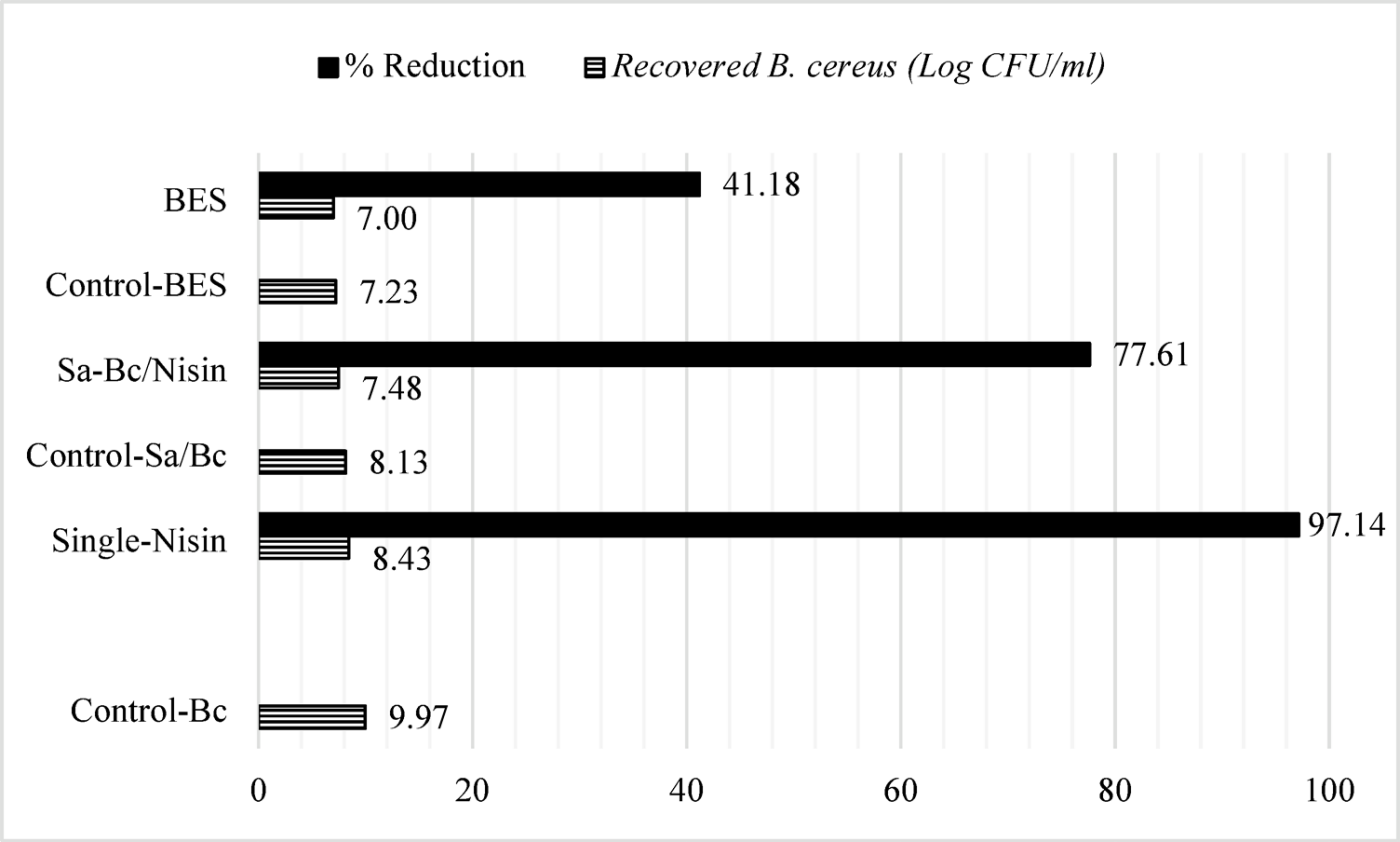
Influence of Nisin/ EDTA on the poly-microbial- included *B. cereus*.

The response of *B. cereus* was different toward Nisin/ EDTA as presented in fig. 5 which shows the maximum density as 9.97 Log CFU/ mL at the single state without preservation (Control). By addition of Nisin/ EDTA, the single-cultured *B. cereus* slowed down its growth to 8.43 Log CFU/ mL (96.64% growth reduction). Introducing *S. aureus* significantly (*P* < 0.05) decreased the effect of Nisin/ EDTA which lowered *B. cereus* growth from 8.13 to 7.48 Log CFU/ mL (77.61% growth reduction. Within the tertiary (*Sa-Bc-Ec*) system, there was a further limiting in the effect of Nisin/ EDTA that just lowered *B. cereus* from 7.23 to 7.00 Log CFU/ mL (41.18% growth reduction).

Response of the gram-negative bacterium; *Escherichia coli* was also reported. The behavior of *E. coli* within the Poly-microbial system is significantly distinguishable from the other two gram-positive partners; *S. aureus* and *B. cereus*, and results were shown in fig, 6. For *E. coli*, the maximum growth (9.1 Log CFU/ mL) was achieved in the single culture without addition of Nisin/ EDTA. Addition of Nisin/ EDTA decreased the estimated growth of the single-cultured *E. coli* to 7.70 Log CFU/ mL (97.14% growth reduction). When combined with *S. aureus*, *E. coli* growth significantly dropped from 8.63 to 8.00 Log CFU/ mL (77.61% growth reduction). Within the tertiary (*Sa-Bc-Ec*) system, the addition of Nisin/ EDTA was noted not to make a significant difference in the count of *E. coli*.

**Fig. 6:**
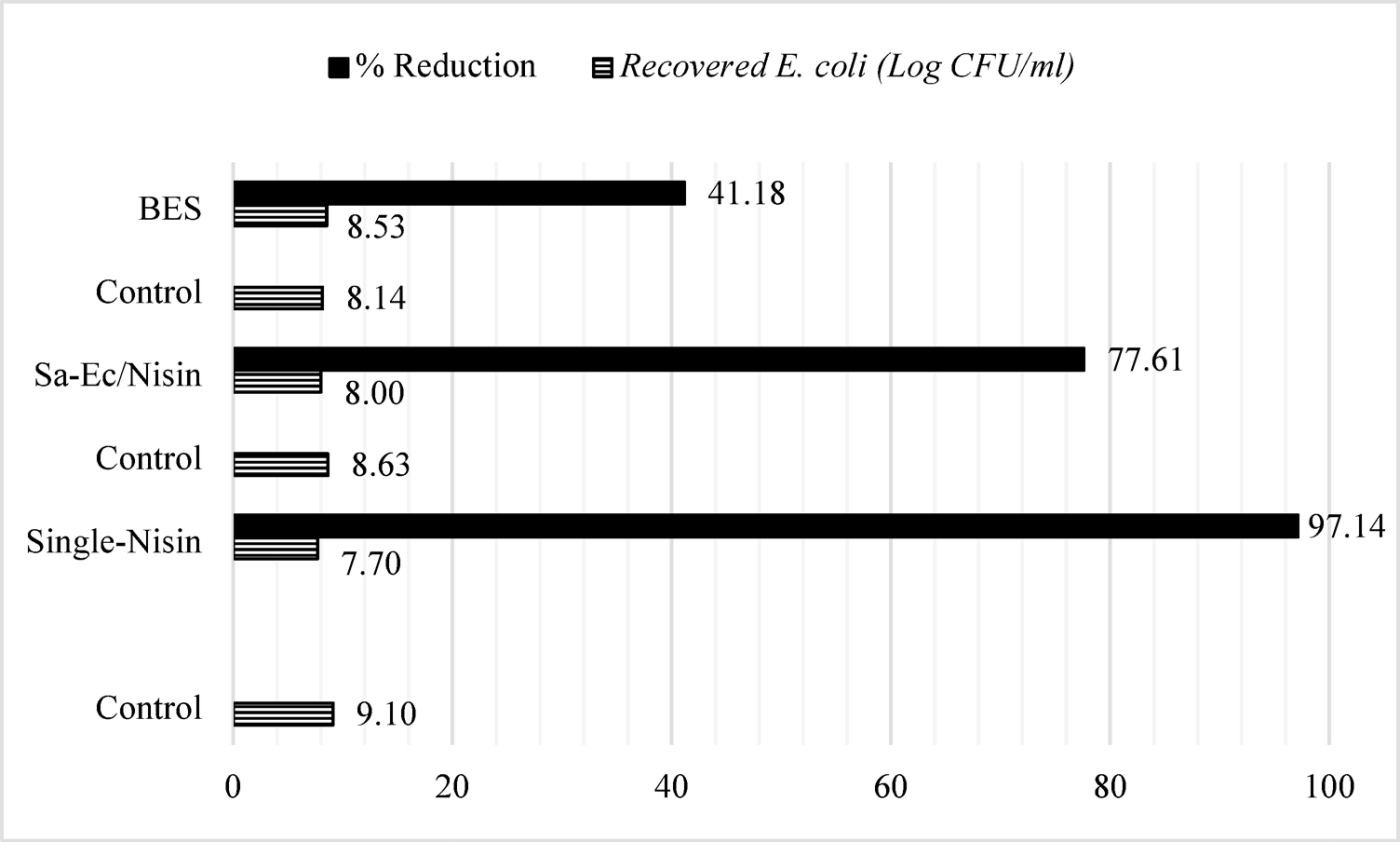
The influence of Nisin/ EDTA on *E. coli* within a poly-microbial culturing.

### In-*Situ* experiment to evaluate the role of Nisin/EDTA a real dairy product

Estimating the effect of nisin and EDTA in a cheese batch as real dairy model. Microbiological status was assessed in the tertiary state (*Sa-Bc-Ec*) either without addition of preservative (Untreated growth), or with preservation (Nisin/EDTA-treated growth). Responses were presented in Table (2).

**Table 2:** Evaluating the role of Nisin/ EDTA in controlling the population of a poly-microbial system.

Microbiological components	Microbial count (Log CFU/ g)								
	0 time	3 days		6 days		9 days		14 days	
		Control	Nisin-EDTA	Control	Nisin-EDTA	Control	Nisin-EDTA	Control	Nisin-EDTA
*B. cereus*	3.35 ± 0.03	3.14 ± 0.05^A^	2.84 ± 0.03^B^	4.83 ± 0.03^A^	2.65 ± 0.03 ^B^	5.34 ± 0.03^A^	2.15 ± 0.03 ^B^	5.67 ± 0.03^A^	2.15 ± 0.03 ^B^
*S. aureus*	3.10 ± 0.07	4.72 ± 0.07 ^A^	2.59 ± 0.07 ^B^	4.58 ± 0.07^A^	2.40 ± 0.07 ^B^	5.09 ± 0.07^A^	1.90 ± 0.07 ^B^	5.42 ± 0.07^A^	1.90 ± 0.07 ^B^
*E. coli*	3.03 ± 0.05	4.65 ± 0.06 ^A^	2.52 ± 0.05 ^B^	4.51 ± 0.05^A^	2.33 ± 0.05 ^B^	5.02 ± 0.05^A^	1.83 ± 0.05 ^B^	5.35 ± 0.05^A^	1.83 ± 0.05 ^B^
Enterobacteriaceae	3.13 ± 0.02	4.8 ± 0.02 ^A^	2.62 ± 0.02 ^B^	4.6 ± 0.02^A^	2.43 ± 0.02 ^B^	5.1 ± 0.02 ^A^	1.93 ± 0.02 ^B^	5.45 ± 0.02^A^	1.93 ± 0.02 ^B^
Mold and yeast	ND	ND	ND	ND	ND	ND	ND	ND	ND
AMC	3.40 ± 0.01	5.2 ± 0.24 ^A^	2.90 ± 0.01 ^B^	4.9 ± 0.01^A^	2.71 ± 0.01 ^B^	5.4 ± 0.01 ^A^	2.20 ± 0.01 ^B^	5.72 ± 0.01^A^	2.20 ± 0.01 ^B^

Mean ± Std. deviation; sharing a letter within the same raw at a particular storage period means no significance.

Beginning with about 3 Log CFU/g, each gram of the untreated cheese showed rising in populations as 3.14, 4.72, 4.65 Log CFU/ g of *B. cereus*, *S. aureus*, and *E. coli* respectively after 3 days of cold storage. Density of these target microbes kept rising to reach (4.83, 4.58, and 4.51 Log CFU/ g), (5.34, 5.09, and 5.02 Log CFU/ g), and (5.67, 5.42, and 5.35 Log CFU/ g) after 6 days, 9 days, and 14 days respectively. Counts of both Enterobacteria and the aerobic mesophilic bacteria also raised as a result to maximally reach 5.45 and 5.72 Log CFU/ g respectively. Inclusion of Nisin/EDTA significantly caused a significant decrease of the (*Sa-Bc-Ec*) microbes to record (2.84, 2.59, and 2.52 Log CFU/ g), (2.65, 2.40, and 2.33 Log CFU/ g), and (2.15, 1.90, and 1.83 Log CFU/ g) after 3 days, 6 days, and 9 days respectively. No change was observed after 14 days (*P* < 0.05). Also, mold and yeast did not record a detectable growth along the storage period.

## Discussion

By measuring the reduction in *S. aureus* growth, it seemed to negatively affected by co-culturing with *E. coli*. In this case (*Sa-Ec*), some of metabolites that possess antimicrobial activity were over-produced. These possible inhibitors include Hydroxyurea that raised from 1.71% to 5.22%, Methane nitroso that raised from 0% to 5.42%, Propylcyclobutene that raised from 0% 12.21%, and 2.,5-dimethyl Pyrazine that raised from 0% to 7.65%. As reviewed, all of the last-mentioned metabolites have a considerable antibacterial effect that thought to contribute to *S. aureus* count reduction^35^. The increased production of these substances may be driven by “*the interference competitive interaction*” in which, one competitor microorganism produces antagonistic factors that harms or kills the other competitor as reviewed by^52^. Similar explanation was reviewed by Raffatellu^53^ who cited that is so-called “contact-dependent inhibition system”. Medveďová and Valík^54^ and Medveďová et al.^55^ concluded that growth of *S. aureus* 2064 was inhibited in the presence of *E. coli* BR and attributed this inhibition either to faster nutrients consumption of *E. coli* or to the production of metabolites with anti-staphylococcal properties e.g. lactic acid, citric acid and other organic acids.

As concluded from fig. 1, there is no significance between the binary (*Sa-Bc*) and the tertiary (*Bc-Ec-Sa*) systems in decreasing the count of *B. cereus* by 95.91% and 94.66% respectively. This means that growth reduction is mainly attributed to the effect of *S. aureus*. In the binary (Sa-Bc) system, it was noted that Methane nitroso reached the maximum percent (8.28%), 2,5-dimethyl Pyrazine also increased maximally to 13.68%. Both compounds are reported as strong antibacterial agents^36^. Furthermore, *B. cereus* inhibition within the tertiary (*Bc-Ec-Sa*) system appears to be caused by the severe production of 1,1-bis (bromanyl)-2-chloranyl-2-fluoranyl-cyclopropane (55.2%) and Propylcyclobutane (33.78%). They two compounds were defined by their good antibacterial activity. Huang^56^ investigates the inhibitory effects of pyrazine volatiles derived from *Bacillus velezensis* on the growth and mycotoxin production of *Fusarium graminearum* and reported the inhibitory effect of pyrazine of *F. graminearum *growth by 41.2% at a concentration of 500 µg/L and considerably reduced zearalenone (ZEN) production by 85.4%. 2,5-dimethylpyrazine and 2,3,5-trimethylpyrazine were the pyrazine derivative detected with relatively high peak areas.

Following the response of *E. coli* (Fig. 2), its growth was affected by the binary (*Ec-Sa*) system at minimum level reducing 66.67% of growth, while maximally reduced within the tertiary (*Bc-Ec-Sa*) system to 96.66%. The minimal effect appears correlated to the increased synthesis of Hydroxyurea (5.22%), Methane, nitroso (5.42%), Propylcyclobutane (12.21%), and 2,5-dimethyl Pyrazine (7.65%). The maximal growth reduction appears correlated to the severe production of antibacterials; 1,1-bis (bromanyl)-2-chloranyl-2-fluoranyl-cyclopropane (55.2%) and Propylcyclobutane (33.78%).

El-Toukhy et al.^57^ reported the presence of *E. coli*,* B. cereus* and *S. aureus* in milk and milk products with 47.5%, 3.3%, and 70.8% of total samples collected from Qalubiya governorates in Egypt. Gundogan and Avci^58^ studied the occurrence of *E. coli*, *S. aureus* and *B. cereus* in raw milk, white cheese and ice cream samples collected in Turkey and reported the highest contamination were detected in raw milk with 70%, 60% and 48% for *B. cereus*, *E. coli* and *S. aureus* respectively. It is worth to mention that the detected *B. cereus*, *E. coli* and *S. aureus* exhibit resistance to some antibiotics such as ampicillin, penicillin and Trimethoprim/sulfamethoxazole and susceptible to cefotaxime. Also; the incidence of Multi-drug resistance pathogens in raw milk and some milk products were reported by Ashraf et al.^59^ with 69.64%, 12.5% and 16.7% for *E. coli*,* S. aureus* and *B. cereus* respectively in the tested samples.

## Conclusion

Interpreting microbial interactions is crucial for developing effective food preservation strategies, enhancing flavor profiles, and ensuring food safety and quality. In poly-microbial systems, growth inhibition among competitors can be attributed to both substrate competition and the production of inhibitory metabolites. Our findings indicate that the presence of *S. aureus* in binary or tertiary systems reduces the growth of other competing microorganisms. Notably, metabolites such as hydroxyurea and butylated hydroxytoluene are potential contributors to the antibacterial properties and require further validation studies. By understanding microbial interactions, we can improve food safety and identify suitable preservation methods, ultimately benefiting both consumers and producers. Furthermore, this knowledge can inform the development of novel food preservation techniques that are both effective and sustainable.

## Data Availability

All data generated or analyzed during this study are included in this published article.
